# Supplementary material for: A Salmonella enterica serovar Typhimurium genome-wide CRISPRi screen reveals a role for type 1 fimbriae in evasion of antibody-mediated agglutination
Source: Infect Immun. 2025 Apr 10;93(5):e00574-24. doi: 10.1128/iai.00574-24 (PMC12070745; doi:10.1128/iai.00574-24)
Supplement: Supplemental material — Fig. S1 to S4; Tables S1 to S3; Legends. [file iai.00574-24-s0005.pdf]

Supplementary Information

A *Salmonella enterica* serovar Typhimurium Genome-wide CRISPRi Screen Reveals a Role for Type 1 Fimbriae in Evasion of Antibody-Mediated Agglutination

Samantha K. Lindberg[1,2], Graham G. Willsey[2], and Nicholas J. Mantis [1,2*]

[1], Department of Biomedical Sciences, University at Albany School of Public Health, Albany, NY, USA; [2], Division of Infectious Diseases, Wadsworth Center, New York State Department of Health, Albany, NY, United States of America

*Correspondence: Dr. Nicholas J. Mantis <nicholas.mantis@health.ny.gov>

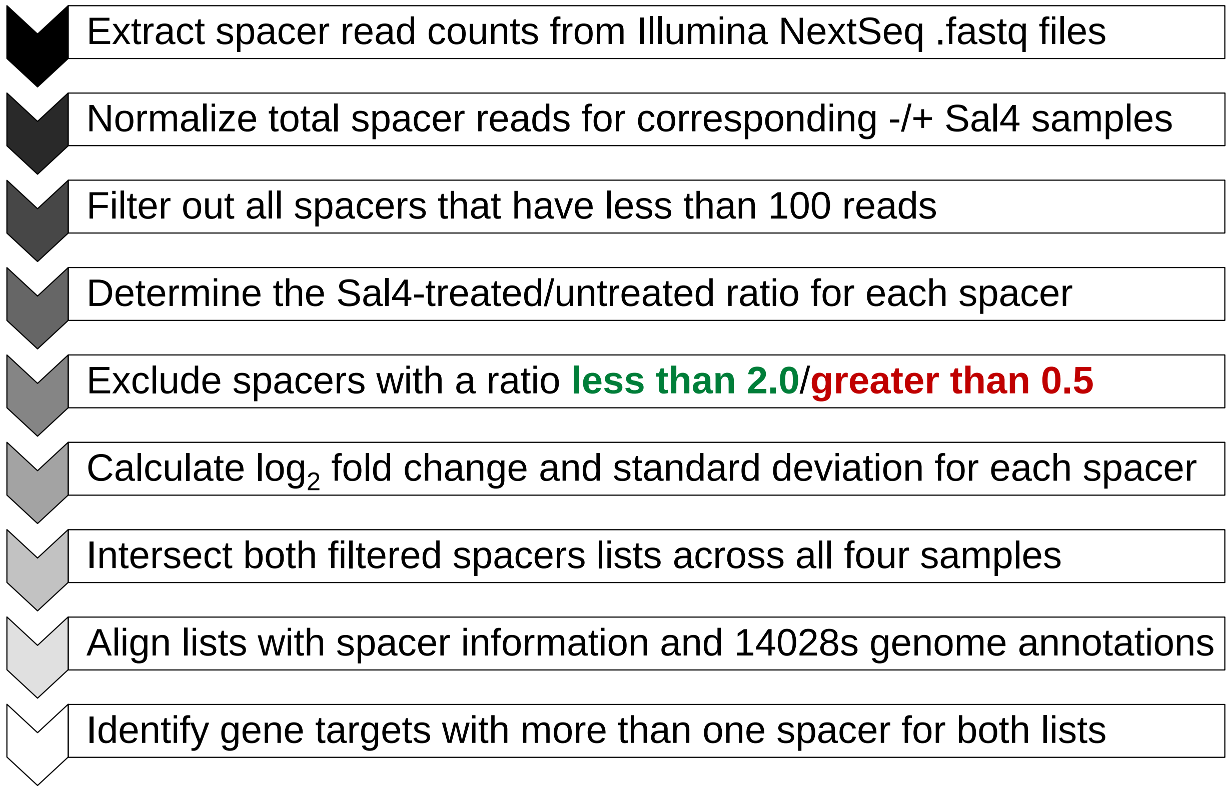

Supplementary Figure 1: Summary of the CRISPRi screen data analysis process. Spacer frequency data for each sample (included in Dataset S2) was analyzed using a custom R script (https://github.com/MantisLab-WadsworthCenter/Salmonella-Typhimurium-CRISPRi-library-analysis) to generate Dataset S4.

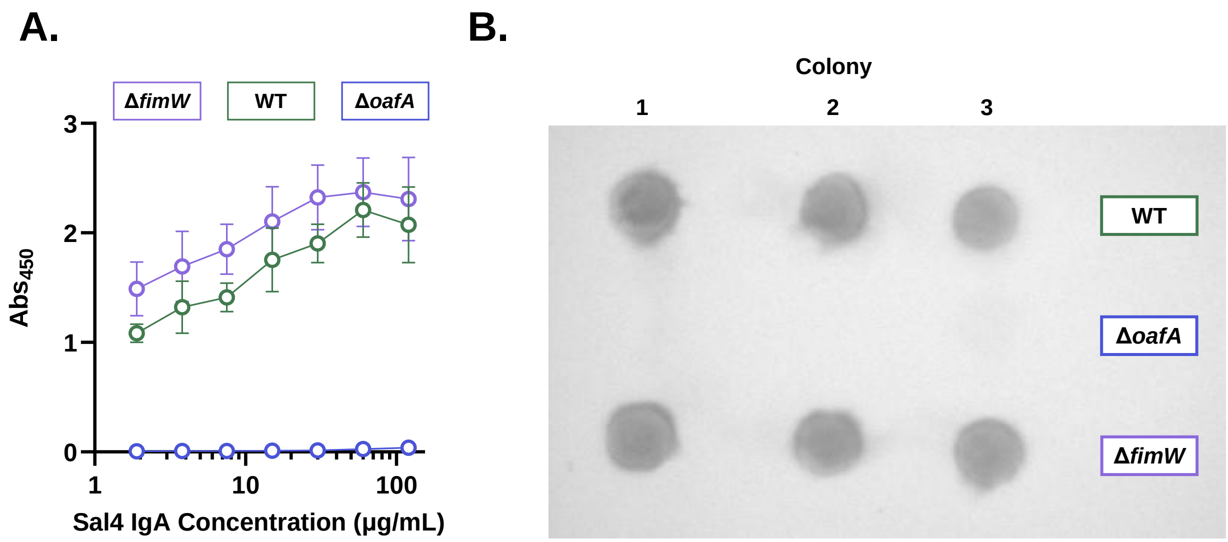

Supplementary Figure 2: Sal4 IgA binds to the *fimW* mutant as measured by ELISA and O5-antigen dot blot. (A) Mid-log phase cultures of WT (SL174; green circles; O5+), Δ*fimW* (SL164; purple circles, O5+), and Δ*oafA* (SL180; blue circles; O5–) were washed in PBS, standardized to an OD_{600} value of 1.0, and incubated in a clear flat-bottom 96-well plate in a fume hood until the culture media had evaporated. Plates were incubated in blocking solution overnight at 4°C, then washed with 0.1% PBS-T, and incubated with Sal4 IgA for 1 h. Plates were washed again, incubated with HRP-conjugated anti-IgA secondary antibodies, and finally developed with TMB substrate prior to reading absorbance at 450 nm (Abs_{450}) using a SpectraMax iD3 plate reader. Data represents two biological replicates averaged from three technical replicates. (B) Representative image of a nitrocellulose membrane spotted with subcultures of the indicated strains. Sal4 binding to STm was detected using a goat anti-mouse IgA-HRP (alpha-chain specific) secondary antibody followed by addition of TMB substrate, as detailed in the Materials and Methods. Each dot for each strain represents an individual colony and the dot blot assay was performed in triplicate.

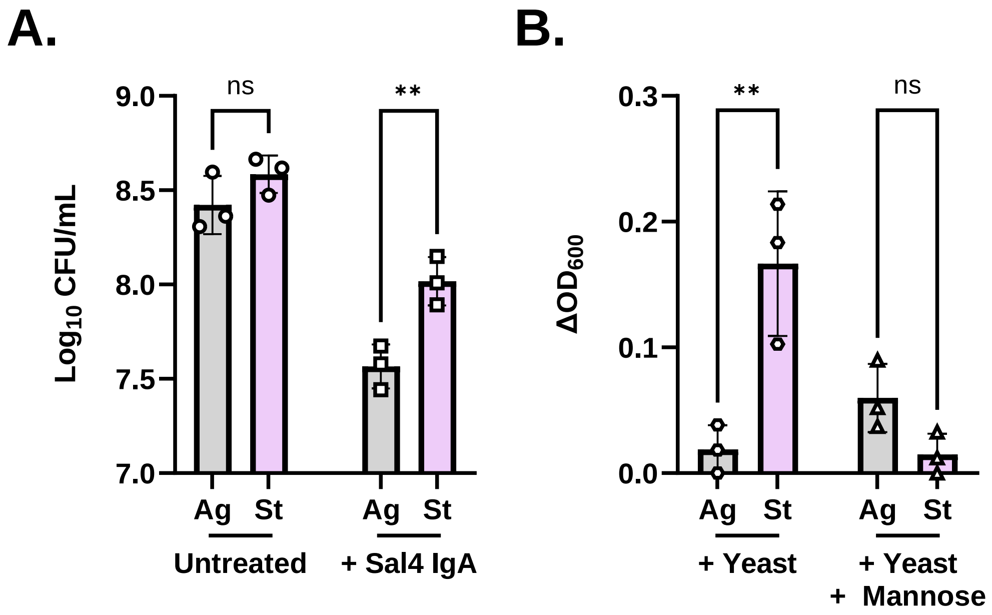

Supplementary Figure 3: Induced T1F expression reduces Sal4-mediated agglutination of WT STm. A single colony of WT STm (SL174) was used to inoculate two separate 5 mL cultures of LB + Kan and then incubated either with agitation (Ag [225 rpm], light grey bars) or statically (St; light purple bars) at 37°C for 48 h. Both cultures were diluted 1:1000 in fresh media after 24 h. (A) After 48 h, the shaking and static cultures were diluted 1:50 or 1:25, respectively, and then grown to mid-log phase, washed in PBS, and either left untreated (circles) or treated with 15 µg/mL of Sal4 IgA (squares). After 2 h of treatment, the top of the supernatant was collected and plated on LB agar to measure CFUs. (B) After 48 h, the shaking and static cultures were centrifuged, resuspended in LB, and concentrated to an OD_{600} of 2.0. Cultures were mixed with yeast (final concentration: 10 mg/mL) in the presence (triangles) and absence (hexagons) of 3% mannose in a 12-well plate and the optical density of the wells at 600 nm (OD_{600}) was measured via spectrophotometry. For both panels, data was obtained from three biological replicates with error bars representing the standard deviation of the mean. Statistical significance was determined by two-way ANOVA followed by Šídák's multiple comparisons test. Asterisks (**) indicate $p < 0.01$ and ns = not significant.

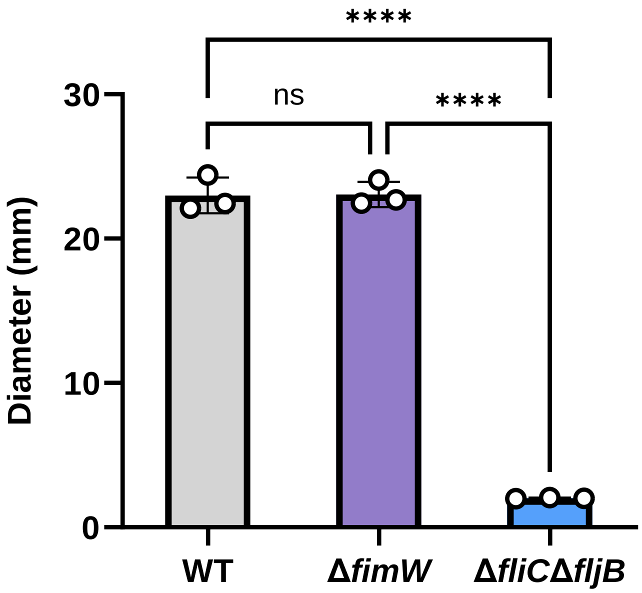

Supplementary Figure 4: STm WT and Δ*fimW* are similarly motile in 0.3% swim agar. Plates of 0.3% LB agar were stab inoculated with 1.0 µL of overnight cultures of WT (SL174), Δ*fimW* (SL164), and Δ*fljB*Δ*fliC* (SL204) and then incubated at 37°C for 4.33 h. Plates were then imaged and the diameter (mm) of the bacterial migration was measured from the plate image using Fiji (version 2.9.0). Data represents three biological experiments each averaged from three technical replicates. Statistical significance was determined by ordinary one-way ANOVA with Tukey's multiple comparisons test. Asterisks (****) indicate $p < 0.0001$ and ns = not significant.

Table S1: Bacterial strains used in this study

Strain	O-Ag	Genotype	Reference[1]
Derivatives of *Salmonella* Typhimurium 14028s			
GGW377	O5	wild type 14028s	ATCC
SJF10	**O4**	*zjg8101::kan oafA126::Tn10d-Tc fkpA-lacZ;* Kan^R	(1)
SL061	O5	Δ*cas3::thyA* (CRISPRi library); Carb^R	
SL094	O5	GGW377 + pKD46; Carb^R	(2)
SL118	**O4**	SJF10 + pBAD24-EV; Kan^R, Carb^R	
SL163	O5	Δ*cheR::kan*; Kan^R	
SL164	O5	Δ*fimW::kan*; Kan^R	
SL165	O5	Δ*invA::kan*; Kan^R	
SL166	O5	Δ*kbl::kan*; Kan^R	
SL167	O5	Δ*mgtBC::kan*; Kan^R	
SL168	O5	Δ*ompR-envZ::kan*; Kan^R	
SL170	O5	Δ*yhfL::kan*; Kan^R	
SL171	O5	Δ*yidYZ::kan*; Kan^R	
SL172	O5	GGW377 (WT) + pTn7; Carb^R	(2)
SL174	O5	*attTn7::*$P_{A1/04/03}$*-lacZ-kan* (WT-*lacZ*) ; Kan^R	(2)
SL180	**O4**	Δ*oafA::kan*; Kan^R	(2)
SL184	O5	Δ*yfeA::kan*; Kan^R	
SL186	O5	Δ*yfiN::kan*; Kan^R	
SL202	O5	Δ*flhC::kan*; Kan^R	(2)
SL204	O5	Δ*fljB::kan* Δ*fliC::gent*; Kan^R, Gent^R	(2)
SL214	O5	GGW377 + pBAD24-EV; Kan^R, Carb^R	
SL216	O5	GGW377 + pBAD24-*fimW*; Kan^R, Carb^R	
SL218	O5	GGW377 + pBAD24-*fimZ*; Kan^R, Carb^R	
SL220	O5	Δ*ompC::kan*; Kan^R	
SL239	O5	*attTn7::kan*; Kan^R, Carb^R	
SL253	O5	SL164 (Δ*fimW::kan)* + pBAD24-EV; Kan^R, Carb^R	
SL255	O5	SL164 (Δ*fimW::kan*) + pBAD24-*fimW*; Kan^R, Carb^R	
SL257	O5	SL174 (WT-*lacZ*) + pBAD24-EV; Kan^R, Carb^R	
SL283	O5	Δ*fimA::kan*; Kan^R	
SL289	O5	SL289 (Δ*fimA::kan*) + pBAD24-EV; Kan^R, Carb^R	
SL291	O5	SL289 (Δ*fimA::kan*) + pBAD24-*fimW*; Kan^R, Carb^R	
Escherichia coli			
DH5α F'I^q	-	F′ *proA*$^+$*B*$^+$*lacI*^{q} Δ(*lacZ*)*M15 zzf::Tn10* (Tet^R) / *fhuA2*Δ(*argF-lacZ*)*U169 phoA glnV44 Φ80*Δ(*lacZ*)*M15 gyrA96 recA1 relA1 endA1 thi-1 hsdR17*; Tet^R	NEB

[1]Strains were generated in this study unless otherwise indicated.

Table S2: Plasmids used in this study

Plasmid	Description	Reference[1]
pKD13	R6Kγ ori; Kan^R	(3)
pKD46	arabinose-inducible λ Red recombinase genes; Carb^R	(3)
pTn7	arabinose-inducible Tn7 transposase; Carb^R	(4)
pUC18-R6k-mTn7-*kanR*	R6Kγ ori; mini-Tn7 integration vector; Kan^R	(5)
pBAD24-EV	Arabinose-inducible, pBR322 ori; Carb^R	(6)
pFimW	pBAD24 with *fimW* insertion at XbaI and HindIII sites; Carb^R	
pFimZ	pBAD24 with *fimZ* insertion at XbaI and HindIII sites; Carb^R	

[1]Plasmids were generated in this study unless otherwise indicated.

Table S3: Oligonucleotide primers used in this study

Primer Name	Primer Sequence (5' to 3')
**Primers used to generate and validate Lambda Red recombination mutant strains**	
cheR_KO_scrnF	CCGATACCTTTCGTCTGGTTCGCATAGA
cheR_lambda_F	CGCGCCCGTTGTACTTTGAATGTGATTAAGAAGGCGCTTGTGTAGGCTGGAGCTGCTTCG
cheR_lambda_R	GCGGAATCATCAACTGACAATACCCTGATTTTACTCATTAATTCCGGGGATCCGTCGACC
fimA_lambda_R	GTTGAGGCGCCTCCCTTCCCTGGCGTTCCCTGACGGATAATTCCGGGGATCCGTCGACC
fimW_KO_scrnF	AGGCCGCCAGTATTTAGAAAATCAAT
fimW_lambda_KO_F	TTTCACCATGATTCACCTGCCGTGTAGGATATTTTTTTTGTGTAGGCTGGAGCTGCTTCG
fimW_lambdaKO_R	TGAGATATTTCGTAAGCCTTGTAAAAAGTTAAGTGAGTTAATTCCGGGGATCCGTCGACC
invA_KO_scrnF	TGCGTTATTGCTTAGTCATAAAGAACATGCATCC
invA_lambda_F	AAAAGCTGTCTTAATTTAATATTAACAGGATACCTATATGTGTAGGCTGGAGCTGCTTCG
invA_lambda_R	ATCCAAATGTTGCATAGATCTTTTCCTTAATTAAGCCCTAATTCCGGGGATCCGTCGACC
kanR_R	GAGCGAGCACGTACTCGGATGG
kbL_lambda_F	TAATATGTGCTGAAATTTGCCCAGTCTGGAGAATCGCATGTGTAGGCTGGAGCTGCTTCG
kbL_lambda_R	TCCGCTTTCAGTTTGGATAACGCTTTCATCTTACATCCTAATTCCGGGGATCCGTCGACC
kbL_lambda_scrn_F	CGTGAGGATACGCGTGATTTATGCTGC
mgtBC_lambda_F	TGTGCTAAATATAGCACGTACTTATTCTTCCAGAAAAATGTGTAGGCTGGAGCTGCTTCG
mgtBC_lambda_R	TCGGGTGAGCGATTCATCTGGGCGATCCTCAAACATTATAATTCCGGGGATCCGTCGACC
mgtBC_lambda_scrnF	TTTCCTCCGCCGTTAACACGACGC
ompC_lambda_F	AAAAAAGCAATAAAGGCATATAACAGAGGGTTAATAACTGTGTAGGCTGGAGCTGCTTCG
ompC_lambda_R	AAAGGGCCCGCAGGCCCTTTAGCAACATCTTTTGCTGATAATTCCGGGGATCCGTCGACC
ompC_lambda_scrnF	GCCGACTGGTTAATGAGGGTTA
ompR-envZ::lamba_F	ACACTTACATTTGTTGCGAACCTTTGGGAGTACAGACATGTGTAGGCTGGAGCTGCTTCG
ompR-envZ::lamba_R	CGGCGTTGAGAAGAAAGGGAGGGTAATACCTCCCTTTCTAATTCCGGGGATCCGTCGACC
ompR-envZ_KO_scrnF	ACGGGGTATAACGTGATCGTCCCGA
T1F_operon_lambda_F	GGATGCCGAAACCGGGTGTGTGTAATTCAAGGGAAATCCGTGTAGGCTGGAGCTGCTTCG
T1F_operon_scrn_F	ATAGCATCGGGCGGCATAAT
yfeA::kanR_F	ATTCATGCGCCTTATATAATGATGTGAGCATTAAAGCATGTGTAGGCTGGAGCTGCTTCG
yfeA::kanR_R	CAGAAACCGGGGAGCTATCCCCGGTTTTTTATGCCGCTAATTCCGGGGATCCGTCGACC
yfeA_KO_scrnF	CTTGCATGGCATGCAAGAGGTCAGC
yfiN::kanR_F	AAATCCAGAAGTATTAATGCTTGCACGGAATCAAAAGCTAATTCCGGGGATCCGTCGACC

yfiN::kanR_R		GGTCTCAACGCTGAGTCAGAAACGGCCAGGCCCGTTCCTGTGTAGGCTGGAGCTGCTTCG
yfiN_KO_scrn		CTGTACTACCTGCGTGAGCGTACTGGTA
yhfL_KO_scrnF		CGCTGAAAGCCGTTTACGAAGCC
yhfL_lamba_F		CGGTTTTTTTGTATCTGCGCAGTCGTTTCTATTATTGATGTGTAGGCTGGAGCTGCTTCG
yhfL_lamba_R		TCCACCAGTAGTGCTCGTTTTATCAACAAGGATTTTGATAATTCCGGGGATCCGTCGACC
yidYZ_lamda_F		CCCCGTATTCTTAGCGCCACACTTTTCGTGAGGCCGCTTGTGTAGGCTGGAGCTGCTTCG
yidYZ_lamda_R		TTCTGGTAAGAGGAAGGTTAATTTATCAACGCAGGTGGTAATTCCGGGGATCCGTCGACC
yidYZ_lamda_scrnF		CAGGGTCAATCTGGCCGGGATCTC
**Primers used to generate modified pBAD24 plasmids**		
fimW_XbaI_F		GCTCTAGATGGGAATTAAGGCCGCCAGT
fimW_HindIII_R		CCCAAGCTTCATCATTGTGGCAGCGTTA
fimZ_HindIII_R		CCCAAGCTTATGCGACCTTCCTGATCAA
fimZ_XbaI_F		GCTCTAGATCAACAGGGAGGTCTCATTC
pBAD24_seq_F		GGGACCAAAGCCATGACAAA
pBAD24_fimW_seq_R		CGCTGAACCAATCATCATCC
pBAD24_fimZ_seq_R		AAAGGCGGGCACCATGATAT
**Ultramers used to amplify and barcode spacer fragment pools for Next-Generation Sequencing**		
**Primer Name**	**Illumina Adapter**	**Primer Sequence (5' to 3')**
JW10363	N502	AATGATACGGCGACCACCGAGATCTACACCTCTCTATTCGTCGGCAGCGTCAGATGTGTATA AGAGACAGTCCTAGGTATAATGCTAGCATAAACCG
JW10365	N505	AATGATACGGCGACCACCGAGATCTACACGTAAGGAGTCGTCGGCAGCGTCAGATGTGTAT AAGAGACAGGTCCTAGGTATAATGCTAGCATAAACC
JW10366	N582	AATGATACGGCGACCACCGAGATCTACACATTGGCACTCGTCGGCAGCGTCAGATGTGTAT AAGAGACAGGTCCTAGGTATAATGCTAGCATAAACC
JW10368	N587	AATGATACGGCGACCACCGAGATCTACACAGCATCTGTCGTCGGCAGCGTCAGATGTGTAT AAGAGACAGTAGCTCAGTCCTAGGTATAATGCTAGC
JW10369	N589	AATGATACGGCGACCACCGAGATCTACACAGATCGTCTCGTCGGCAGCGTCAGATGTGTAT AAGAGACAGTAGCTCAGTCCTAGGTATAATGCTAGC
JW10370	N506	AATGATACGGCGACCACCGAGATCTACACACTGCATATCGTCGGCAGCGTCAGATGTGTAT AAGAGACAGCAGCTAGCTCAGTCCTAGGTATAATGC
JW10330	N701	CAAGCAGAAGACGGCATACGAGATTAAGGCGAGTCTCGTGGGCTCGGAGATGTGTATAAGA GACAGTCTCATCCGCCAAAACAGC

Legend for Supplementary Data Files:

- **Data set S1** (.xlsx): Spacer assignment data for the CRISPRi library. Sheet 1 lists the spacer sequence and associated information (PAM sequence, genome coordinate, associated gene and locus tag, and spacer ID).
- **Data set S2** (.xlsx): Combined spacer frequencies for each sample in the CRISPRi screen. Following .fastq processing using the python script available at https://github.com/wade-lab/Salmonella-CRISPRi, the raw .txt files were imported into Excel for further analysis. The spreadsheet includes 8 tabs listing each library spacer sequence and the associated frequency for each sample (E = biological experiment number, P = passage number, U = untreated condition, Sal4 = Sal4-treated condition, and R = technical replicate number) and one tab ('combined') with spacer sequences and frequency data for all 8 samples. Raw .fastq files for each sample are available on ArrayExpress under the accession ID E-MTAB-14834.
- **Data set S3** (.xlsx): Annotated genome information and operon prediction for STm 14028s (RefSeq Assembly ID: GCF_000022165.1) obtained from the genome2D NCBI RefSeq mirror database (URL: http://genome2d.molgenrug.nl/).
- **Data set S4** (.xlsx): CRISPRi screen analysis data. As outlined in Figure S1, a custom analysis (https://github.com/MantisLab-WadsworthCenter/Salmonella-Typhimurium-CRISPRi-library-analysis) was used to process the raw spacer frequency data (Dataset S2) into the resulting spreadsheet. The spreadsheet has four tabs, 'final_enriched' and 'final_reduced' includes the enriched/de-enriched spacer lists with log2 fold change (average and for each matched sample) and standard deviation values along with associated spacer assignment and annotated genome information (RefSeq Assembly ID: GCF_000022165.1). The 'enriched_mult' and 'reduced_mult' sheets lists all genes from the final enriched/reduced lists with more than one associated enriched/de-enriched spacer.

References:

1. Forbes SJ, Eschmann M, Mantis NJ. 2008. Inhibition of *Salmonella enterica* Serovar Typhimurium Motility and Entry into Epithelial Cells by a Protective Antilipopolysaccharide Monoclonal Immunoglobulin A Antibody. Infect Immun 76:4137–4144. https://doi.org/10.1128/IAI.00416-08

2. Lindberg SK, Willsey GG, Mantis NJ. 2023. Flagellar-based motility accelerates IgA-mediated agglutination of *Salmonella* Typhimurium at high bacterial cell densities. Front Immunol 14:1193855. https://doi.org/10.3389/fimmu.2023.1193855

3. Datsenko KA, Wanner BL. 2000. One-step inactivation of chromosomal genes in *Escherichia coli* K-12 using PCR products. Proc Natl Acad Sci USA 97:6640–6645. https://doi.org/10.1073/pnas.120163297

4. Jasinska W, Manhart M, Lerner J, Gauthier L, Serohijos AWR, Bershtein S. 2020. Chromosomal barcoding of E. coli populations reveals lineage diversity dynamics at high resolution. Nat Ecol Evol 4:437–452. https://doi.org/10.1038/s41559-020-1103-z

5. Choi K-H, Gaynor JB, White KG, Lopez C, Bosio CM, Karkhoff-Schweizer RR, Schweizer HP. 2005. A Tn7-based broad-range bacterial cloning and expression system. Nat Methods 2:443–448. https://doi.org/10.1038/nmeth765

6. Guzman LM, Belin D, Carson MJ, Beckwith J. 1995. Tight regulation, modulation, and high-level expression by vectors containing the arabinose PBAD promoter. J Bacteriol 177:4121–4130. https://doi.org/10.1128/jb.177.14.4121-4130.1995
